# Investigation Into the Current Situation and Analysis of the Factors Influencing Extrauterine Growth Retardation in Preterm Infants

**DOI:** 10.3389/fped.2021.643387

**Published:** 2021-04-30

**Authors:** Ting Zhao, Hui-Ming Feng, Bayier Caicike, Yan-Ping Zhu

**Affiliations:** Department of Neonatology, The First Affiliated Hospital of Xinjiang Medical University, Urumqi, China

**Keywords:** preterm infants, extrauterine growth retardation, EUGR, risk factors, retrospective, intrauterine growth retardation

## Abstract

**Objective:** This study aims to explore the occurrence of extrauterine growth retardation (EUGR) in preterm infants with a gestational age of <34 weeks, at discharge, and the factors influencing the occurrence of EUGR.

**Method:** A retrospective analysis of 691 preterm infants with a gestational age of less than 34 weeks, born in our hospital over the past 3 years. At discharge, the growth indicators head circumference, weight, and length were used to divide the infants into an EUGR group (*n* = 255) and the non-EUGR group (*n* = 436). The occurrence of EUGR and its influencing factors were then analyzed.

**Results:** Of the 691 preterm infants evaluated for inclusion in the study, 255 cases (36.9%) met the requirements of EUGR at discharge. The different growth indicators used, i.e., weight, length, and head circumference, classified the infants differently. The incidence of EUGR using these measures was 30.2% (209), 27.9% (193), and 23.2% (161), respectively. The results of a univariate analysis showed that gestational age, birth weight, intrauterine growth retardation (IUGR), maternal gestational hypertension, age at which the infant commenced feeding, duration of the application of an invasive ventilator, length of hospital stay, nosocomial infection, respiratory and gastrointestinal diseases, symptomatic patent ductus arteriosus, and the early onset of neonatal sepsis were correlated with the occurrence of EUGR. Further logistic multivariate regression analysis revealed that low gestational age, low birth weight, complicated IUGR, respiratory distress syndrome, and necrotizing enterocolitis were independent risk factors for EUGR in preterm infants with a gestational age <34 weeks.

**Conclusion:** In preterm infants with a gestational age <34 weeks in our hospital, there is a high incidence of EUGR, which is affected by factors such as the gestational age, birth weight, IUGR, respiratory distress syndrome, necrotizing enterocolitis, and other factors.

## Introduction

American researchers, Clark et al., first proposed the definition for extrauterine growth restriction (EUGR) in preterm infants in 2003, i.e., newborns with the growth indicators (weight, length, and head circumference) less than the 10th percentile of the average growth indicators in infants of the same gestational age (1). Studies have found that preterm infants with EUGR are at increased risk of developing respiratory disease after their discharge from the hospital. They are also prone to developing physical dysplasia in the short term and may encounter complications such as adolescent hypertension, metabolic and endocrine abnormalities, and serious neurological development problems in the long term, which may affect quality of life (2–5). Therefore, EUGR is still a serious problem in preterm infants. It is important to investigate the factors influencing EUGR in preterm infants and to reduce its occurrence (6). Our hospital is an autonomous, region-level care center for critical newborns, and admits many critically ill newborns, especially preterm infants. It is important that the treatment of preterm infants not only improve their survival rate, but also improve their long-term prognosis, reduce the incidence of complications, and improve cognitive ability (7). This study, conducted a retrospective analysis of the occurrence of EUGR at discharge in preterm infants with a gestational age of <34 weeks, born in our hospital over the past 3 years. Based on observation at our hospital and relevant professional knowledge, together with knowledge gleaned from relevant domestic and foreign literature (8), risk factors that might lead to EUGR in preterm infants were identified. This research aims to provide a reference for the clinical prevention of EUGR.

## Materials and Methods

### Study Subjects

Preterm infants with a gestational age of <34 weeks, who had been admitted to the neonatal intensive care unit (NICU) of the First Affiliated Hospital of Xinjiang Medical University within the past 3 years, were enrolled as study subjects. The inclusion criteria were as follows: (1) neonates born in our hospital or transferred to our department within 24 h of birth with gestational age <34 weeks, who survived for more than 7 days and (2) neonates with complete medical records. The exclusion criteria were as follows: (1) neonates who died during hospitalization or were discharged abnormally or (2) neonates with diseases of the surgical system, severe developmental abnormalities or chromosomal abnormalities, or genetic metabolic diseases.

### Methods

Data were collected and placed in a clinical database for preterm infants for a research project undertaken by the Children's Hospital of Fundan University. These data were used for a cluster randomized controlled trial named “Reduction of infection in neonatal intensive care units using the evidence-based practice for improving quality” (9). The data for this study were drawn from this database. The data came from a single center of the hospital. Data were retrospectively collected. These data included general data at admission and discharge including weight, length, head circumference, gestational age, gender, birth weight, intrauterine growth retardation (IUGR), asphyxia at birth, maternal gestational hypertension, gestational diabetes mellitus, effective administration of maternal hormones, age of commencing feeding, duration of the application of traumatic ventilator, length of hospital stay, the occurrence of nosocomial infection, and the application of a peripherally inserted central catheter; and data about related complications including respiratory distress syndrome, pulmonary hemorrhage, bronchopulmonary dysplasia, feeding intolerance, necrotizing enterocolitis, gastrointestinal hemorrhage, symptomatic patent ductus arteriosus, pulmonary hypertension, metabolic disorders, early onset of neonatal sepsis, intracranial hemorrhage, neonatal anemia, etc. According to the EUGR diagnostic criteria, the infants were divided into an EUGR group and a non-EUGR group based on the growth parameters, head circumference, weight, and length, at the time of discharge.

### Diagnostic Criteria for EUGR and IUGR

Infants with a weight, length, or head circumference below the 10th percentile of the average growth parameters, adjusted by the gestational age at discharge, were defined as having EUGR (1). Infants with a birth weight less than the 10th percentile of the growth curve of the corresponding gestational age were defined as having IUGR. The reference standard was the reference value of the percentiles of the weight distributions of newborns of different gestational ages from 25 provinces, municipalities, and autonomous regions in China (10).

### Data Collection

A retrospective, case-control study method was adopted to collect the clinical data and the complications experienced by the study subjects.

### Statistical Analysis

The SPSS 22.0 software was used for data analysis. The countable data were expressed as percentages and the measurable data were expressed as mean ± standard deviation ($\bar{x}$ ± s). The χ^2^ test or *t-*test was adopted for univariate analysis. An analysis of the influencing factors was conducted using logistic regression analysis. *P* < 0.05 was considered statistically significant.

## Results

### The Incidence of EUGR at Discharge in Preterm Infants

A total of 972 preterm infants with gestational age <34 weeks were admitted during the study period. A total of 281 preterm infants who died during hospitalization, or had severe congenital developmental abnormalities, inherited metabolic diseases, chromosomal abnormalities, or automatic discharge were excluded from the study. A total of 691 subjects were enrolled in the study. The incidence of EUGR was 36.9% (255) at the time of discharge. Of these subjects, 53.3% (136) had measurements less than the 10th percentile of neonates of the same gestational age in all three areas, i.e., weight, head circumference, and length. When considering each measurement criterion separately, i.e., weight, length, and head circumference, the incidence of EUGR was 30.2% (209), 27.9% (193), and 23.2% (161), respectively, as shown in Table 1.

**Table 1:** The percentage of preterm infants in the EUGR and non-EUGR groups evaluated by weight, length, or head circumference (*n* = 691).

**Group**	**Evaluated by weight**	**Evaluated by length**	**Evaluated by head circumference**	**Evaluated by weight, length, or head circumference**
EUGR	209 (30.2%)	193 (27.9%)	161 (23.2%)	255 (36.9%)
Non-EUGR	482 (69.8%)	498 (72.1%)	530 (76.8%)	436 (63.1%)

The 691 cases were classified according to their gestational age, and the difference in the incidence of EUGR was statistically significant between the age groups (*P* < 0.05). Preterm infants with a gestational age of <30 weeks had the highest incidence of EUGR (78.2%), and those with a gestational age of 32–34 weeks had the lowest incidence of EUGR (21.2%). The details are shown in Table 2.

**Table 2:** Comparison of the incidence of EUGR at discharge in 691 preterm infants with a gestational age < 34 weeks among different gestational age groups [*n* (%)].

**Different gestational age groups**	**Cases (*n* = 691)**	**The incidence of EUGR (*n* = 255)**
<30 weeks	110	86 (78.2)
30–32 weeks	322	114 (35.4)
32–34 weeks	259	55 (21.2)
χ^2^		108.1
*P*		<0.001

The 691 cases of the preterm infants were also classified by weight, and the difference in the incidence of EUGR between the weight groups was statistically significant (*P* < 0.05). Preterm infants with a weight <1,500 g had the highest incidence of EUGR (97.6%), and those with a weight of more than 2,500 g had the lowest incidence of EUGR (7.1%). The details are shown in Table 3.

**Table 3:** Comparison of the incidence of EUGR at discharge in 691 preterm infants with a gestational age < 34 weeks among different weight groups [*n* (%)].

**Different weight groups**	**Cases (*n* = 691)**	**The incidence of EUGR (*n* = 255)**
<1,500 g	124	121 (97.6)
1,500–2,500 g	539	132 (24.5)
>2,500 g	28	2 (7.1)
χ^2^		247.482
*P*		<0.001

### Univariate Analysis of the Influencing Factors of EUGR

The average gestational age and average birth weight in the EUGR group were lower than those in the non-EUGR group (*P* < 0.05). The incidence of complicated IUGR, gestational hypertension, age of commencement of feeding, duration of the application of traumatic ventilator, length of hospital stay, and nosocomial infection in the EUGR group were all higher than those in the non-EUGR group (*P* < 0.05), as shown in Table 4.

**Table 4:** Comparison of the general characteristics at discharge in preterm infants with a gestational age < 34 weeks between the non-EUGR group and EUGR group.

**Related factors**	**Non-EUGR group** ** (*n* = 436)**	**EUGR group** ** (*n* = 255)**	***t/χ*^2^**	***P***
Gestational age (Week, $\bar{x}$ ± s)	32.4 ± 1.2	30.6 ± 1.6	−15.546	0.000
Male [*n* (%)]	259 (59.4)	134 (52.5)	3.082	0.079
Birth weight (g, $\bar{x}$ ± s)	1704.2 ± 624.1	1440.2 ± 420.8	−9.547	0.001
Incidence of IUGR [*n* (%)]	65 (14.9)	88 (34.5)	35.86	0.000
Incidence of asphyxia at birth [*n* (%)]	90 (20.6)	69 (27.1)	3.739	0.053
Maternal gestational hypertension [*n* (%)]	113 (25.9)	107 (42.0)	19.415	0.000
Gestational diabetes mellitus [*n* (%)]	54 (12.4)	21 (8.2)	2.864	0.091
Maternal effective administration of hormone [*n* (%)]	321 (73.6)	178 (69.8)	1.170	0.279
Age of start feeding (d, $\bar{x}$ ± s)	3.2 ± 1.8	4.3 ± 2.6	5.544	0.042
Duration of the application of traumatic ventilator (Day)	5.8 ± 5.1	9.6 ± 3.2	−5.926	0.001
Length of hospital stay (Day)	11.4 ± 5.9	18.3 ± 14.8	7.08	0.000
Nosocomial infection [*n* (%)]	4 (0.1)	14 (5.5)	13.261	0.000
Application of PICC [*n* (%)]	145 (33.3)	103 (40.4)	3.56	0.059

### Univariate Analysis of the Related Complications in the EUGR Group

This study suggests that the incidence of complicated respiratory system diseases (respiratory distress syndrome, pulmonary hemorrhage, and bronchopulmonary dysplasia), gastrointestinal diseases (feeding intolerance, necrotizing enterocolitis, and gastrointestinal hemorrhage), symptomatic patent ductus arteriosus, and the early onset of neonatal sepsis in the EUGR group was significantly higher (*P* < 0.05) than in the non-EUGR group, as shown in Table 5.

**Table 5:** Comparison of the complications in preterm infants with a gestational age < 34 weeks between the non-EUGR group and EUGR Group.

**Complication**	**Non-EUGR group** ** (*n* = 436)**	**EUGR group** ** (*n* = 255)**	**χ^**2**^**	***P***
Respiratory distress syndrome [*n* (%)]	42 (9.86)	51 (20.0)	14.847	0.000
Pulmonary hemorrhage [*n* (%)]	3 (0.1)	7 (2.7)	4.773	0.029
Bronchopulmonary dysplasia [*n* (%)]	10 (2.3)	17 (6.7)	8.195	0.004
Feeding intolerance [*n* (%)]	17 (3.9)	25 (9.8)	9.827	0.002
Necrotizing enterocolitis [*n* (%)]	12 (2.8)	18 (7.1)	7.185	0.007
Gastrointestinal hemorrhage [*n* (%)]	4 (0.9)	9 (3.5)	5.947	0.015
Symptomatic patent ductus arteriosus [*n* (%)]	21 (4.8)	22 (8.6)	4.004	0.045
Pulmonary hypertension [*n* (%)]	8 (1.8)	10 (3.9)	2.761	0.097
Metabolic disorder [*n* (%)]	318 (72.9)	196 (76.7)	1.302	0.254
Early-onset of neonatal sepsis [*n* (%)]	21 (4.8)	24 (9.4)	5.581	0.018
Intracranial hemorrhage [*n* (%)]	340 (78.0)	188 (73.7)	1.617	0.204
Neonatal anemia [*n* (%)]	252 (57.8)	165 (64.7)	3.208	0.073

### Multivariate Logistic Regression Analysis of the High-Risk Factors of EUGR

The statistically significant variables from the univariate analysis were introduced into the logistic multivariate regression analysis and the results revealed that low gestational age, low birth weight, IUGR, respiratory distress syndrome, and necrotizing enterocolitis were independent risk factors for EUGR. The results are shown in Table 6.

**Table 6:** Multivariate logistic regression analysis of the risk factors for the occurrence of EUGR.

	**B**	**S.E**.	**Wald *χ*^2^**	***P***	**OR**	**95% C.I. of EXP(B)**	
						**Lower limit**	**Upper limit**
Constant	−14.491	6.261	5.357	0.021	0.000		
Gestational age	3.224	1.414	5.210	0.023	25.138	1.574	401.565
Birth weight	1.440	0.459	9.843	0.002	4.221	1.717	10.377
IUGR	3.565	1.104	10.434	0.001	35.333	4.062	307.324
Length of stay	0.541	0.226	5.733	0.017	1.718	1.103	2.674
Respiratory distress syndrome	0.788	0.205	14.751	0.000	2.199	1.471	3.286
Necrotizing enterocolitis	2.054	0.968	4.502	0.034	7.799	1.169	52.005

## Discussion

**Figure 1:**
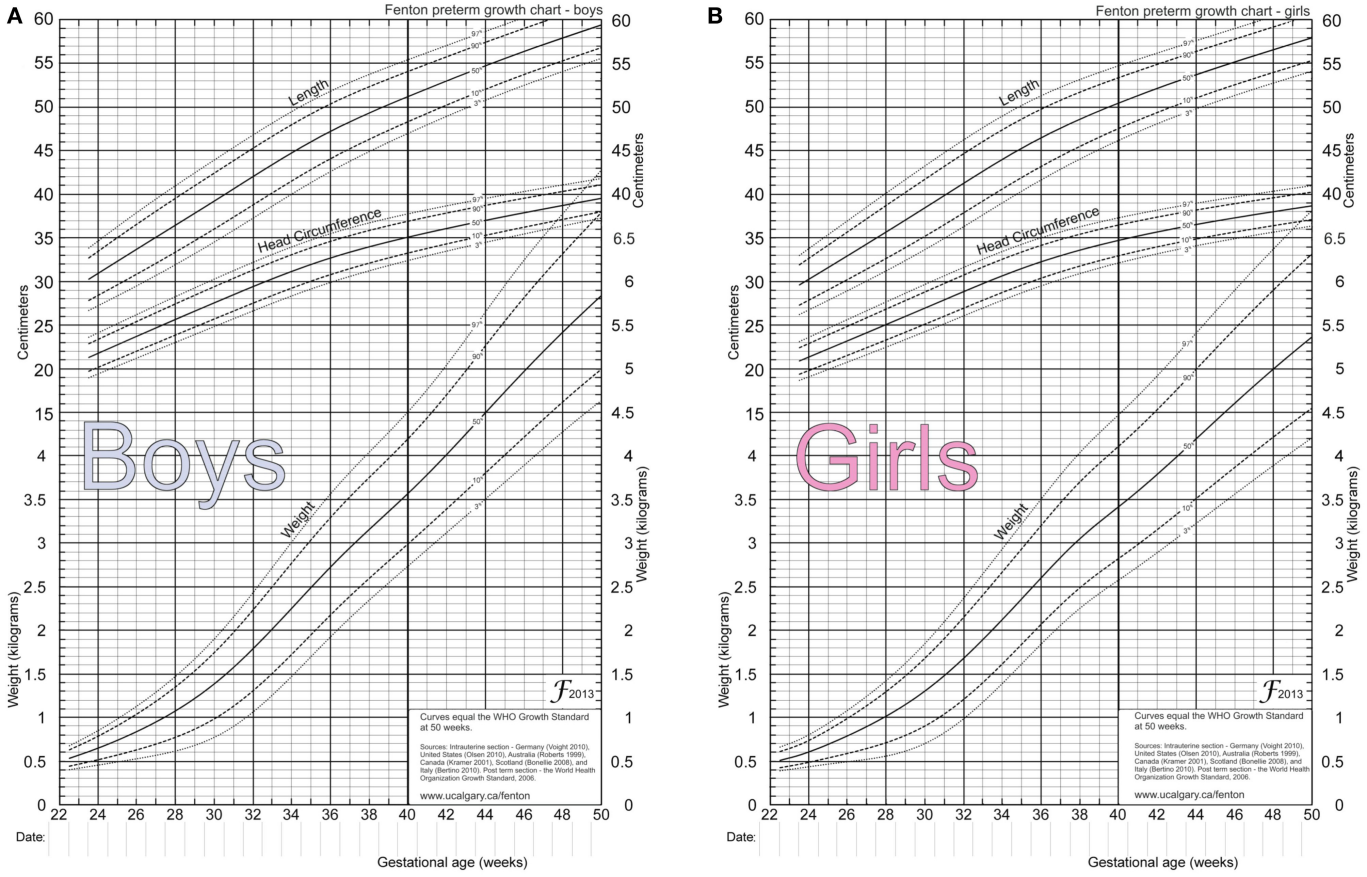
2013 Fenton preterm growth charts for newborns. **(A)** Is the 2013 Fenton preterm growth chart for boys. **(B)** Is the 2013 Fenton preterm growth chart of girls.

Preterm infants account for the highest percentage of inpatients in the NICU in our hospital. In this hospital, in accordance with national trends in the national neonatal specialty, the ability to treat preterm infants has improved rapidly in recent years, and many very preterm infants and infants with very low birth weight survive. However, there are more complications in preterm than in full-term infants, most notably the high incidence of EUGR (5, 11). The results of this study show that the incidence of EUGR in 691 preterm infants with a gestational age of <34 weeks born in our hospital over the past 3 years was 36.9% (255). During hospitalization, the online calculator www.growthcalculator.org is recommended to assess weight gain. It provides an accurate graphic display of the current weight percentile, target weight and its deviation. Fenton 2013 charts may be an alternative to monitor linear and head growth while in the neonatal unit (12) (Figure 1). When assessing the infants using weight, length and head circumference, the incidence of EUGR was 30.2% (209), 27.9% (193), and 23.2% (161), respectively. Of the 255 preterm infants with EUGR, all the growth parameters were lower than the 10th percentile of neonates of the same gestational age in 136 of the cases, which indicates that there is a high probability that a preterm infant with EUGR has values in every growth parameter lower than the 10th percentile. When compared with the data from a developed country, the incidence of EUGR in our hospital was higher. Clark et al. analyzed 24,371 preterm infants with a gestational age of 23–34 weeks hospitalized in 127 NICUs in the United States. On discharge, the infants were evaluated according to weight, length, and head circumference; and the incidence of EUGR based on these measures was 28, 34, and 16%, respectively (1). Radmacher et al. retrospectively analyzed 199 infants with a birth weight ≤ 1,000 g, or a gestational age ≤ 29 weeks, and revealed the incidence of EUGR to be 59.3% (13). Therefore, it has been suggested that there is a high incidence of EUGR in the population of very preterm infants, or infants with very low birth weight. Therefore, this population is worthy of attention. In 2015, the collaboration group of the Nutrition Expert Committee of the Neonatal Professional Committee of the Chinese Medical Doctor Association conducted a multi-center survey of 572 infants with very low birth weight, in 15 hospitals across the country. The incidence of EUGR at discharge was 80.9%, while 63.6% had a weight lower than the P3 percentile. Cai Yueju et al. evaluated the incidence of EUGR in preterm infants according to weight, length, and head circumference and found that the incidence was 26.1, 16.4, and 13.7%, respectively. In the present study, the overall incidence of EUGR in preterm infants was high when compared with the aforementioned studies. The incidence of EUGR in preterm infants with a gestational age of <30 weeks and a birth weight of <1,500 g is particularly high and is far higher than in other countries (14). This suggests that the prevention of EUGR and the treatment of preterm infants with EUGR still requires attention in clinical practice.

It was also found in this study that the prenatal factors related to EUGR in preterm infants were gestational hypertension and IUGR. Complicated IUGR was also found to be an independent risk factor for EUGR. This supports the results of Cai Yueju and Lee et al. (15, 16). Among the maternal factors, premature delivery is largely caused by gestational hypertension, especially eclampsia, which may lead to maternal vasoconstriction, insufficient placental blood flow, low fetal nutritional reserve, and IUGR (17). A randomized clinical study in the United States found that, when compared with conventional milk feeding, high-dose milk feeding could achieve total enteral nutrition in the shortest time possible, increase the rate of weight gain, and reduce the occurrence of EUGR. High-dose milk feeding did not increase the incidence of necrotizing enterocolitis (NEC), bronchopulmonary dysplasia, or other complications (18). Therefore, this part of the population is also the focus of attention.

The present study suggested that low gestational age and low birth weight are independent risk factors for EUGR on discharge. Preterm infants with a low gestational age still have inadequate nutritional status after large quantities of nutritional intake. This results in a significant decrease in weight, a long time to return to the birth weight, and slow weight gain, all of which lead to a long stay in hospital and a greater probability of EUGR (19). Preterm infants with an early onset of neonatal sepsis, respiratory diseases, nosocomial infections, and those requiring the use of an invasive ventilator are in a state of high consumption, increased calorie demand, and excessive protein breakdown. Preterm infants with the additional complication of respiratory disease and symptomatic patent ductus arteriosus may also be faced with restrictions in fluid intake, reduced nutrient intake, and severe negative nitrogen balance soon after birth. These factors also cause and increase in the incidence of EUGR (20, 21). Therefore, adequate nutrition is needed in the early stage after birth to ensure that infants at risk will not lose too much weight, which will help to reduce the incidence of EUGR (22). It was also found that the duration of the application of an invasive ventilator, nosocomial infections, respiratory diseases (respiratory distress syndrome, pulmonary hemorrhage, and bronchopulmonary dysplasia), symptomatic patent ductus arteriosus, and early-onset of neonatal sepsis were high-risk factors for EUGR. Respiratory distress syndrome was identified as an independent risk factor. This study also revealed that the incidence of gastrointestinal diseases such as feeding intolerance, necrotizing enterocolitis, and gastrointestinal hemorrhage was higher in the EUGR group than in the non-EUGR group. The results of the logistic multivariate regression analysis suggested that necrotizing enterocolitis is a risk factor for EUGR. This might be correlated with the impaired intestinal motility, reduced digestion and absorption, impaired intestinal microcirculation perfusion regulation, and impaired intestinal regeneration and healing after NEC, which might affect the development and maturity of the gastrointestinal tract and nutritional intake in preterm infants (23). Active nutrition strategies include using early, active intravenous nutrition, ensuring total enteral nutrition as soon as possible and commencing feeding, with breastfeeding being the preferred method, as early as possible. The use of active nutrition strategies could significantly reduce the incidence of EUGR (24, 25). This view was confirmed in a random cohort study by the University of Bordeaux in France (26).

In summary, to reduce the incidence of EUGR in preterm infants, the focus should be on preterm infants with a low gestational age and a very low birth weight. Clinical management must focus on nutritional supply, which includes starting breastfeeding as soon as possible in combination with active parenteral nutritional support, actively preventing complications, and decreasing the duration of invasive respiratory support, with the aim of closing the gap in extrauterine growth and development.

## Data Availability Statement

The original contributions presented in the study are included in the article/supplementary material, further inquiries can be directed to the corresponding author/s.

## Ethics Statement

The studies involving human participants were reviewed and approved by Ethics Committee of The First Affiliated Hospital of Xinjiang Medical University. Written informed consent to participate in this study was provided by the participants' legal guardian/next of kin.

## Author Contributions

TZ and H-MF: conception and design of the research and writing of the manuscript. TZ and BC: acquisition of data. H-MF: analysis and interpretation of the data. Y-PZ: statistical analysis. Y-PZ and BC: critical revision of the manuscript for intellectual content. All authors contributed to the article and approved the submitted version.

## Conflict of Interest

The authors declare that the research was conducted in the absence of any commercial or financial relationships that could be construed as a potential conflict of interest.
